# Tumor Necrosis Factor Alpha Mediates GABA_**A**_ Receptor Trafficking to the Plasma Membrane of Spinal Cord Neurons *In Vivo*

**DOI:** 10.1155/2012/261345

**Published:** 2012-03-12

**Authors:** Ellen D. Stück, Randolph N. Christensen, J. Russell Huie, C. Amy Tovar, Brandon A. Miller, Yvette S. Nout, Jacqueline C. Bresnahan, Michael S. Beattie, Adam R. Ferguson

**Affiliations:** ^1^Brain and Spinal Injury Center (BASIC), Department of Neurological Surgery, University of California, San Francisco, CA 94110, USA; ^2^Biology Department, Coe College, Cedar Rapids, IA 52402, USA; ^3^Department of Neuroscience, The Ohio State University, Columbus, OH 43210, USA; ^4^Department of Neurological Surgery, Emory University, Atlanta, GA 30322, USA; ^5^Animal and Veterinary Sciences Department, California State Polytechnic University, Pomona, CA 91768, USA

## Abstract

The proinflammatory cytokine TNF*α* contributes to cell death in central nervous system (CNS) disorders by altering synaptic neurotransmission. TNF*α* contributes to excitotoxicity by increasing GluA2-lacking AMPA receptor (AMPAR) trafficking to the neuronal plasma membrane. *In vitro*, increased AMPAR on the neuronal surface after TNF*α* exposure is associated with a rapid internalization of GABA_A_ receptors (GABA_A_Rs), suggesting complex timing and dose dependency of the CNS's response to TNF*α*. However, the effect of TNF*α* on GABA_A_R trafficking *in vivo* remains unclear. We assessed the effect of TNF*α* nanoinjection on rapid GABA_A_R changes in rats (*N* = 30) using subcellular fractionation, quantitative western blotting, and confocal microscopy. GABA_A_R protein levels in membrane fractions of TNF*α* and vehicle-treated subjects were not significantly different by Western Blot, yet high-resolution quantitative confocal imaging revealed that TNF*α* induces GABA_A_R trafficking to synapses in a dose-dependent manner by 60 min. TNF*α*-mediated GABA_A_R trafficking represents a novel target for CNS excitotoxicity.

## 1. Introduction

Gamma amino butyric acid type A receptors (GABA_A_Rs) are a major source of fast inhibitory synaptic transmission in the CNS and thus play a crucial role in regulating neuronal networks. Altering the number of GABA_A_Rs in a synapse through rapid receptor trafficking therefore can have a major inhibitory impact on neuronal excitability [1–3]. Trafficking of GABA_A_Rs to and from the plasma membrane has been shown to depend on neuronal activity [4–7]. GABA_A_R redistribution is also mediated by a variety of neuromodulatory substances such as hormones and cytokines. For example, insulin and PI3 kinase cause rapid insertion of GABA_A_Rs into the plasma membrane increasing the amplitude of miniature inhibitory postsynaptic currents (mIPSCs) [8, 9].

Tumor necrosis factor alpha (TNF*α*), a proinflammatory cytokine that is constitutively expressed following spinal cord injury (SCI), has been shown to alter receptor trafficking of GABA_A_Rs to and from the cell surface *in vitro* [10]. *In vivo,* TNF*α* levels are increased as part of the inflammatory response following SCI and various other nervous system disorders, contributing to secondary cell death [11–13]. TNF*α* has been shown to specifically promote trafficking of glutamate-receptor-2-(GluA2-) lacking AMPA receptors (AMPARs) to the plasma membrane of spinal neurons inducing excitotoxicity *in vivo* [13]. Blocking TNF*α* action pharmacologically reduces AMPAR trafficking and cell death after SCI, suggesting that modulation of AMPARs is a major mechanism by which TNF*α* promotes cell death in CNS disease [13].

The aim of the current study is to assess *in vivo *the effect of TNF*α* on GABA_A_R trafficking to the plasma membrane of spinal neurons. We modeled the inflammatory response associated with SCI by TNF*α* nanoinjection into the spinal cord to establish whether GABA_A_Rs are exocytosed onto or endocytosed from the plasma membrane. By determining the direction of trafficking, we can then infer whether trafficking of GABA_A_Rs is exacerbating or attenuating TNF*α*-mediated excitotoxicity. GABA_A_Rs were identified and quantified based on the presence of the gamma-2 (*γ*2) subunit as studies show that this subunit is critical for postsynaptic clustering of GABA_A_Rs and the vast majority of GABA_A_Rs are composed of subunits *α* and *β* combined with *γ*2 [14, 15]. We used a combination of biochemical fractionation and laser scanning confocal microscopy followed by iterative deconvolution and automated image analysis to evaluate GABA_A_R levels in the plasma membrane and at synapses after TNF*α* nanoinjection into the spinal parenchyma. Whole membrane fractionation and quantitative western blotting showed a trend towards increased membrane GABA_A_R protein levels within 60 min of TNF*α* nanoinjection delivery, but these results did not reach significance. Confocal data revealed increased GABA_A_Rs at synaptic sites following TNF*α* nanoinjection, underscoring the subcellular specificity of GABA_A_R localization. Confocal image findings also suggest that there is a nonlinear dose-dependent relationship between TNF*α* and GABA_A_R trafficking.

## 2. Materials and Methods

### 2.1. Animals

Female Long-Evans rats, 77- to 87-d-old, were housed in pairs (*N* = 30) with *ad libitum* access to food and water. All procedures were nonsurvival surgeries performed under deep anesthesia. All possible steps were taken to avoid unnecessary suffering. All experimental procedures followed the National Institutes of Health guidelines and were approved by the Institutional Animal Care and Use Committees at The Ohio State University and the University of California, San Francisco.

### 2.2. TNF*α* Nanoinjection

**Figure 1:**
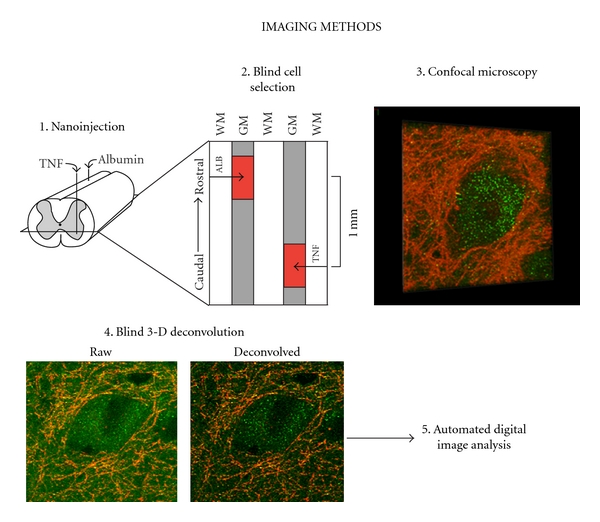
Overview of *in vivo *nanoinjection methods for confocal imaging experiments. (1) Subjects (*n* = 4) received a single TNF*α* injection dose (0.01, 0.1, or 1 *μ*M) as well as a contralateral control injection of albumin. (2) Injection sites were 1 mm apart along the rostrocaudal axis. Injection sites were localized using FluoroRuby (depicted as red regions). (3) Within injection sites, large ventral motor neurons were selected under wide-field fluorescence in a blinded fashion by using the characteristic pattern of presynaptic synaptophysin outlining the plasma membrane to identify cells. (4) Confocal z-stacks were deblurred by 3D blind iterative deconvolution (AutoQuant). Panels depict a z-stack before (left) and after (right) deconvolution. (5) Custom macros designed using MetaMorph software (Molecular Devices) were used to quantify the number of fluorescently-labeled receptor puncta on the plasma membrane. WM: white matter; GM: gray matter.

Nanoinjections (35 nl) were delivered stereotactically into the T9-10 ventral horn as described in Hermann et al., 2001, by applying compressed air micropressure to pulled glass pipettes (tip diameter, 30 *μ*m; 30° bevel; Radnoti). An albumin injection served as a control due to its similarity in molecular weight to rat recombinant TNF*α* (R&D systems). For imaging experiments, subjects (*n* = 4) received a dose of TNF*α* (0.01, 0.1, or 1 *μ*M) and an injection of albumin on the contralateral ventral horn to serve as a within-subject control. These doses of TNF*α* have been found sufficient to increase glutamate-mediated excitotoxicity in the spinal cord, but insufficient to cause cell death [11]. The dye FluoroRuby (Invitrogen) was included in each injection solution in order to localize injection sites for high-resolution confocal analyses (Figure 1). Subjects were sacrificed 60 minutes following nanoinjection by transcardiac perfusion with 0.9% saline followed by 4% paraformaldehyde. For biochemical experiments, subjects received four evenly spaced nanoinjections of either TNF*α* (1 *μ*M) or albumin along a 750 *μ*m length of spinal cord (TNF*α*, *n* = 8; vehicle, *n* = 10).

### 2.3. Subcellular Fractionation by Centrifugation

One hour after nanoinjection, the spinal cord was extracted under deep anesthesia; 7.5 mm of the cord was snap-frozen with dry ice and stored at −80°C for later processing. Fractionation procedures were based on prior work with rat spinal cord [13]. The snap-frozen spinal cord was thawed on ice and homogenized with 30 passes of a “Type B” pestle in a Dounce homogenizer (Kontes) with 500 *μ*L of homogenization buffer (10 mM Tris, 300 mM sucrose, Roche miniComplete protease inhibitor, pH = 7.5). The resulting suspension was then passed through a 22 gauge needle five times and centrifuged at 5,000 RCF for 5 min at 4°C. The supernatant (S1) was transferred to a new tube and centrifuged again at 13,000 RCF for 30 min at 4°C. The supernatant (S2) was transferred to a new tube, and the membrane-enriched pellet (P2) was resuspended in 50 *μ*L of PBS containing protease inhibitor. P2 fractions were vortexed and sonicated, and all sample fractions were stored at −80°C.

### 2.4. Protein Assay and Immunoblotting

Sample protein concentration was assayed using BCA (Pierce) and quantified with a plate reader (Tecan; GeNios). P2 fractions from each subject were diluted 1 : 2 with cold Laemmli sample buffer containing 5%  *β*-mercaptoethanol (BioRad), and 20 *μ*g of protein per lane was immediately loaded onto a precast 10% Tris-HCl polyacrylamide gel (BioRad). Sample loading on the gel was performed so that each subject had their own lane, loading order was counterbalanced across injection condition to account for regional variability within the gel, and the experimenter was blind to subject condition. A kaleidoscope ladder (3 *μ*L; BioRad) was loaded to confirm molecular weights. The loaded gel was electrophoresed in SDS running buffer (BioRad; 25 mM Tris, 192 mM glycine, 0.1% SDS, pH = 8.3) for 1 h at 100 volts. The protein was then transferred to nitrocellulose membrane in cold tris-glycine buffer (25 mM Tris, 192 mM glycine, 20% methanol, pH = 8.3). The membrane was blocked for 1 h in Odyssey blocking buffer (Li-Cor) containing 0.1% Tween 20 and then incubated overnight (18 h) in the dark at 4°C in a primary antibody solution containing Odyssey blocking buffer, 0.05% Tween 20, and a rabbit polyclonal anti- GABA_A_ receptor *γ*2 primary antibody (1 : 500; Chemicon, AB5559). The membrane was washed 4 × 5 min with TBS containing 0.1% Tween 20 (TTBS) then incubated for 1 h in a fluorescently labeled secondary antibody solution containing Odyssey blocking buffer, 0.2% Tween 20, and 1 : 30,000 IRDye 680 goat anti-rabbit secondary antibody (Li-Cor) and subsequently washed 4 × 5 min in TTBS, 1 × 5 min in TBS. The membrane was immediately scanned for protein bands using the corresponding 680 nm laser at a scanning intensity of 4 on the Odyssey Infrared Imaging System (Li-Cor). The membrane was then reblocked and reincubated in a primary antibody solution containing 1 : 800 mouse anti-N-Cadherin primary antibody (BD Biosciences, 610920). The membrane was washed and reincubated in a secondary antibody solution containing 1 : 30,000 IRDye 800 goat anti-mouse secondary antibody (Li-Cor). The membrane was washed again and rescanned for protein bands in the 800 nm channel at a scanning intensity of 3.

### 2.5. Quantitative Fluorescence Western Blotting

**Figure 2:**
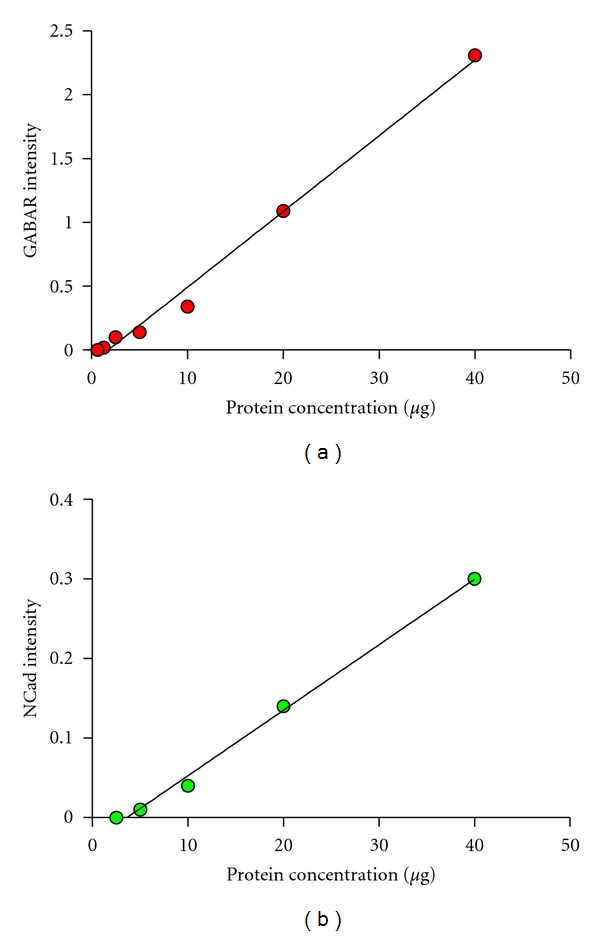
Quantitative Western Blot linear ranges for each protein. (a) GABA_A_R protein band fluorescent intensity corresponding to a protein dilution curve, 680 nm laser at a scanning intensity of 4 (*R* ^2^ = 0.9916). (b) NCad protein band fluorescent intensity corresponding to a protein dilution curve, 800 nm laser at a scanning intensity of 3 (*R* ^2^ = 0.9957).

Although traditional Western Blot analysis using chemiluminescence and densitometry measurements is considered to be merely semiquantitative, we used an established near-infrared labeling and detection technique (Odyssey Infrared Imaging System, Li-Cor) to definitively quantify the intensity of fluorescently labeled protein bands. To ensure that our intensity measurements were truly quantitative, we generated linear ranges for each antibody by plotting band intensity measurement relative to the concentration of protein loaded for a protein dilution curve. Laser scanning intensities for each antibody were selected by determining the laser intensity which yielded the highest linear range, *R* ^2^, of protein band fluorescent intensity from a protein dilution curve of a control sample (Figures 2(a) and 2(b)). The intensity of each fluorescently labeled protein band was quantified using the Odyssey Application Software Version 3.0 (Li-Cor). Background fluorescence was assessed and corrected for using Odyssey Software which determined median pixel densities above and below each protein band and normalized these bands of interest accordingly.

Prior experiments have used the plasma membrane protein N-Cadherin (NCad) to characterize the degree of membrane enrichment in each fraction generated by spinal cord subcellular fractionation [13, 16]. In these studies, western blotting revealed that subcellular fractionation generates P2 samples with a modest enrichment of the plasma membrane as evidenced by the presence of NCad. Neither NCad (*P* = 0.883) nor actin (*P* = 0.610) intensity varied between conditions. We therefore used NCad as a control both for variability in protein loading and in plasma membrane enrichment through subcellular fractionation [16]. GABA_A_R band intensities from each sample were normalized with respect to NCad by dividing GABA_A_R intensity by NCad intensity. All biochemistry was performed in a blinded, counterbalanced fashion. Two independent replications were performed, and the normalized densitometry results were averaged across runs.

### 2.6. Histological Processing

 The 30 mm length of spinal cord centered on the injection site was isolated and postfixed overnight (<18 h) in 4% paraformaldehyde followed by cryoprotection of the tissue in 30% sucrose for 2d. The tissue was then cut into 10 mm blocks, flash-frozen on dry ice, embedded in OCT, and sectioned into 20 *μ*m thick horizontal slices.

### 2.7. Immunohistochemistry

Fixed tissue sections from a full set of experimental conditions were antibody-labeled using a high-throughput staining station (Sequenza; Thermo Scientific). Tissue was blocked and permeabilized with 5% normal goat serum and 0.3% Triton X-100 for 1 h. Sections were incubated in a solution consisting of mouse monoclonal antibody against presynaptic synaptophysin (1 : 200; Millipore MAB5258-50UG) and rabbit polyclonal antibody against GABA_A_ receptor *γ*2 primary antibody (1 : 200; Chemicon, AB5559) overnight at room temperature. Slides were washed with 2 mL PBS then incubated for 1 h at room temperature in a solution containing 1 : 100 Alexa 488 goat anti-rabbit and 1 : 100 Alexa 633 goat anti-mouse secondary antibodies. After washing with 2 mL PBS, slides were coverslipped with Vectashield containing DAPI (4′, 6-diamidino-2-phenylindole; Vector Laboratories). Negative immunolabel control conditions consisted of no primary antibody, and each individual primary with the incorrect secondary. Confocal microscopy of the negative control slides showed no detectable label exceeding threshold.

### 2.8. Confocal Microscopy Sampling and Deconvolution

Large ventral motor neurons (characterized by a diameter >40 *μ*m) were selected using wide-field fluorescence based on the distinctive synaptophysin outline surrounding the plasma membrane (Figure 1). One motor neuron was sampled every 100 *μ*m following the rostrocaudal axis of each ventral horn centered around the FluoroRuby-labeled injection sites. This sampling procedure was performed through horizontal sections of the spinal cord up to 600 *μ*m rostral and caudal to the center of each injection site. In a sampling region of multiple motor neurons, a single motor neuron was chosen at random. A Zeiss 510 META laser scanning confocal microscope (63x objective; NA = 1.4; 2x zoom) was used to generate confocal stacks for large motor neurons. Control tissue was used to optimize filter and laser settings, which were then held constant throughout the experiment. These settings allowed for virtually complete distinction between immunolabels GABA_A_Rs (Alexa 488), synaptophysin (Alexa 633), and FluoroRuby (Texas Red). Confocal z-stacks consisted of 1 *μ*m slices which were oversampled at 0.5 *μ*m z-intervals. AutoQuant software was used to deblur these confocal stacks through the process of 3D blind iterative deconvolution. An iteration number of 3 was determined for GABA_A_R labeling based on a random subset of images and held constant during the experiment. Performing deconvolution on confocal image stacks allowed for greater resolution of receptor puncta than was possible with either technique alone.

### 2.9. Confocal Image Analysis

Images underwent automated image analysis to quantify the number of fluorescently labeled receptor puncta on the plasma membrane exceeding a predetermined pixel threshold based on control tissue. Automated image analysis was performed using custom designed MetaMorph (Molecular Devices) macros. One macro was designed to measure the amount of total GABA_A_R receptor pixels (intra- and extracellular puncta) as well as colocalization of synaptophysin and GABA_A_R pixels (synaptically localized GABA_A_R puncta) in each field of the z-stack. This macro allowed for measurement of GABA_A_R puncta at the level of the neuropil. Another macro quantified fluorescently labeled GABA_A_R puncta on the plasma membrane of motor neurons. First, the macro identified the plane in the z-series with the highest amount of GABA_A_R/synaptophysin colocalization. A blinded researcher supervised the automated plane selection to prevent selection based on staining artifacts. Once a single plane was selected, a blinded researcher identified the plasma membrane of the motor neuron by tracing the synaptophysin-labeled outline of the cell. A 2 *μ*m-thick “image-based subcellular fraction” was produced containing the plasma membrane of the cell from the single plane. From the plasma membrane subcellular image fraction, MetaMorph quantified GABA_A_R pixels (extrasynaptic receptors) and colocalized GABA_A_R/synaptophysin pixels (synaptic receptors).

### 2.10. Data Analysis

Quantitative Western Blot data were analyzed using an analysis of variance (ANOVA). Immunofluorescence data were analyzed using ANOVA, and the experiment was a mixed design (injection side and distance from injection site served as within-subject variables). When appropriate, Tukey's post hoc analyses were used to determine significant differences amongst multiple outcomes within a variable. Total GABA_A_R protein levels were accounted for by ANCOVA, which allowed for the distinction between receptor trafficking versus an increase in total GABA_A_R puncta as a result of receptor synthesis. Significance was established as *P* < 0.05.

## 3. Results

### 3.1. Quantitative Western Blot Detects a Nonsignificant Trend of Increased GABA_**A**_R in Total Membrane Fractions

**Figure 3:**
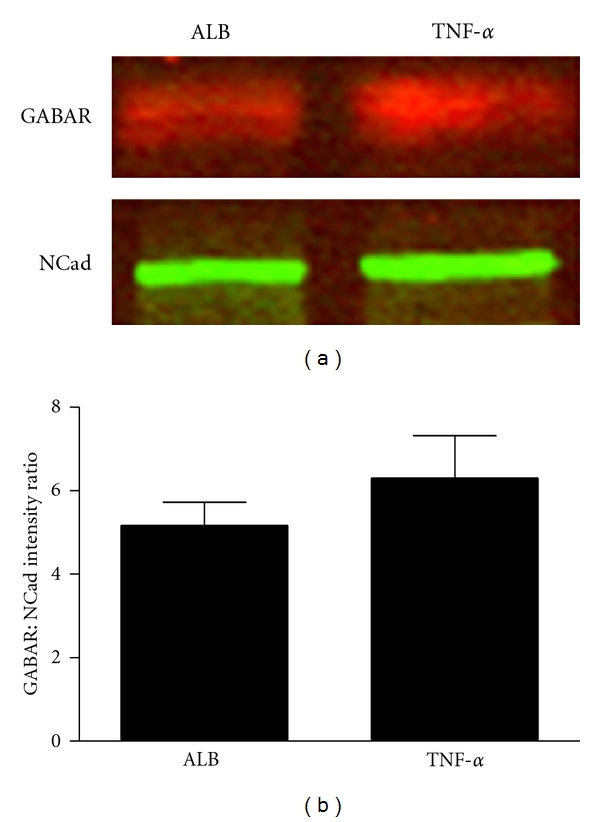
Quantitative western blotting reveals a nonsignificant trend towards an increase in plasma membrane GABA_A_R. (a) Representative examples of Western Blots of membrane-enriched homogenate fractions from an albumin subject and a TNF*α* subject that were run on the same gel. GABA_A_R (red) and plasma membrane protein N-Cadherin (NCad; green) bands were visualized and quantified using the Odyssey IR Imaging System (Li-Cor). (b) Linear intensity quantification of Western Blots of the P2 fraction yields a trend suggesting an increase in GABA_A_R : NCad ratio following TNF*α* injection (*P* = 0.315, *n* = 10 albumin subjects, *n* = 8 TNF*α* subjects). Bars represent group intensity means averaged across 2 Western Blots. Error bars indicate SEM.

Quantitative western blotting was used to generate normalized GABA_A_R intensity ratios (GABA_A_R : NCad) for the membrane-enriched fraction of spinal cord homogenate (Figure 3(a)). These ratios for each subject were generated by running all subjects across two counterbalanced gels. ANOVA revealed no significant difference in total membrane GABA_A_R between injection condition (*P* = 0.315), yet group means reflect a nonsignificant trend towards a higher concentration of GABA_A_Rs in TNF*α*-treated subjects (Figure 3(b)). A crude subcellular fractionation of samples, which in the past has been sufficient to reveal changed receptor levels from TNF*α*-mediated trafficking of AMPARs [13], did not show a significant distinction in GABA_A_R between injection conditions perhaps due to a lack of membrane resolution. Therefore, the high-resolution, low-throughput method of quantitative confocal microscopy was used to elucidate changes in localized receptor trafficking.

### 3.2. Quantitative Confocal Microscopy Reveals That TNF*α* Increases Synaptic and Total GABA_**A**_R in the Neuropil

**Figure 4:**
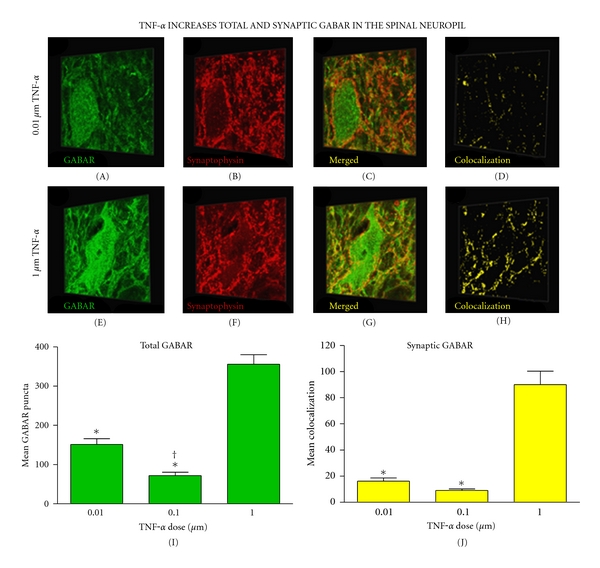
Increased synaptic GABA_A_R expression in the neuropil 60 min after TNF*α* injection. (a)–(h) Three-dimensional representative images of motor neurons demonstrating a dose-dependent increase in synaptic GABA_A_Rs in the neuropil after TNF*α* injection. (i) Quantification of confocal stacks shows a significant increase in total GABA_A_Rs following the highest dose of TNF*α* (*P < 0.001 from middle and lowest doses). An increase in total GABA_A_Rs was also observed in the lowest dose relative to the middle dose (^†^ *P* = 0.015). (j) An increase in synaptic GABA_A_Rs occurred following the highest dose of TNF*α* (*P < 0.001 from middle and lowest doses). Bars represent group means across >800 confocal image stacks (12 subjects, *n* = 4 subjects per group). Error bars reflect SEM. Scale bar, 30 *μ*m.

Automated image analysis of the 3-dimensional neuropil (defined as the entire set of pixels in a confocal z-stack) revealed a dose-dependent increase in synaptic GABA_A_R (colocalized GABA_A_R and synaptophysin pixels) 60 minutes following TNF*α* nanoinjection (*P* < 0.001). Total GABA_A_R in the neuropil increased in a dose-dependent manner (*P* < 0.001) as well (Figure 4). Additionally, there was a significant increase in total GABA_A_R puncta from the middle to the lowest TNF*α* dose (*P* < 0.02) (Figures 4(i) and 4(j)). Studies have shown that GABA_A_Rs can undergo rapid activity-dependent ubiquitination and lysosomal degradation to maintain homeostatic levels of the receptor [15, 17], which could explain the significant decrease in GABA_A_R associated with the middle dose of TNF*α* relative to the lowest dose. It is possible that the low dose of TNF*α* elicited a modest increase in GABA_A_R undetectable to regulatory systems within the neuron, whereas a middle dose of TNF*α* sufficiently increased GABA_A_Rs to the point where they would be downregulated.

An ANCOVA was used to statistically account for the TNF*α*-mediated increase in total GABA_A_R protein in the neuropil, thus isolating the dose-dependent changes in synaptic GABA_A_R attributable to receptor trafficking. After correcting for changes in total GABA_A_R protein, the dose-dependent effect of TNF*α* on synaptic GABA_A_Rs in the neuropil was robustly maintained (*P* < 0.001) indicating that the dose-dependent increase in synaptic GABA_A_R does not depend solely on wholesale protein changes but also relies on receptor trafficking to the synapse. Correcting for variance in total GABA_A_R protein in the neuropil with ANCOVA, revealed no significant interaction of dose by side for synaptic GABA_A_R (*P* = 0.325). Overall, results reveal that TNF*α* dose significantly influenced GABA_A_R receptor trafficking to synaptic sites within the neuropil containing spinal motor neurons.

### 3.3. Analysis of Confocal Microscopic Images Reveals That TNF*α* Increases Extrasynaptic and Synaptic GABA_**A**_R on the Plasma Membrane of Ventral Motor Neurons

**Figure 5:**
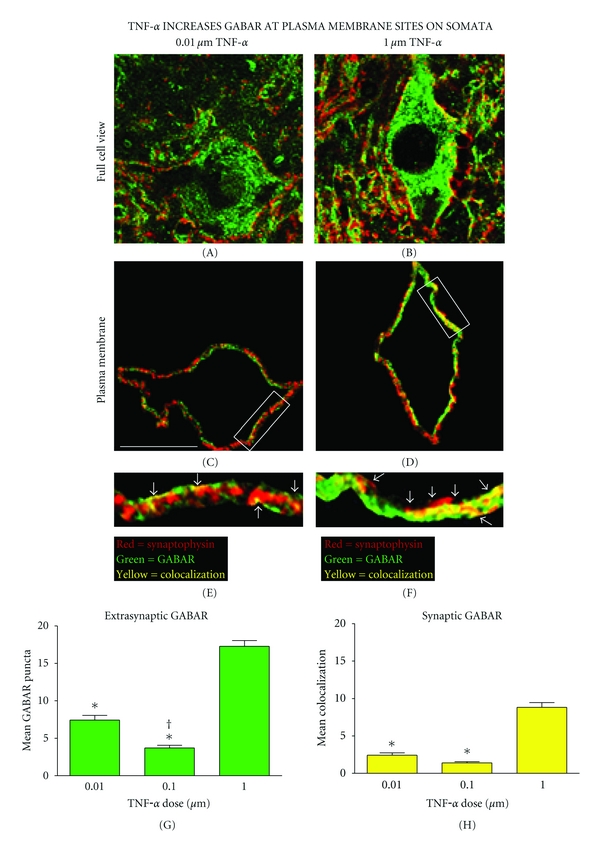
Increased GABA_A_Rs on the plasma membrane of spinal neurons after TNF*α* injection. ((a) and (b)) An image analysis program identified the confocal plane with the greatest colocalization of GABA_A_R and synaptophysin for each motor neuron. ((c) and (d)) A 2 *μ*m wide cutout of the image containing the somatic plasma membrane. ((e) and (f)) Boxed plasma membrane fractions enlarged to demonstrate representative differences in extrasynaptic (green) and synaptic (yellow) GABA_A_R puncta on the plasma membrane following the highest and lowest injections of TNF*α* *in vivo*. ((g) and (h)) The highest dose of TNF*α* yielded a significant increase in extrasynaptic and synaptic GABA_A_Rs on the plasma membrane (extrasynaptic GABA_A_R *P < 0.001 from low and middle dose; synaptic GABA_A_R *P < 0.001 from low and middle dose). Furthermore, the lowest dose of TNF*α* produced a significant increase in extrasynaptic GABA_A_Rs relative to the middle dose (^†^ *P* = 0.002). Bars represent group means across > 800 confocal image stacks (12 subjects, *n* = 4 subjects per group). Error bars reflect SEM. Scale bar, 30 *μ*m.

**Figure 6:**
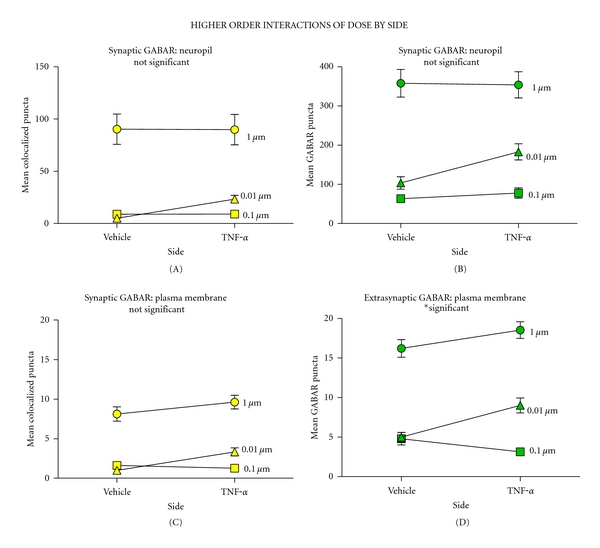
Two-way interactions of drug dose and injection side. ((a)–(d)) There was a significant effect of dose across all outcomes (*P* < 0.001). High doses of TNF*α* had a widespread effect extending onto the contralateral side of the cord such that there was no effect of injection side across any outcome (*P* > 0.05). ((a)–(c)) A 2-factor mixed ANOVA revealed that there was not a significant interaction between dose and side on total GABA_A_R on the neuropil, synaptic GABA_A_R on the neuropil, or plasma membrane (*P* > 0.05); yet there was a significant effect of interaction for extrasynaptic GABA_A_R on the plasma membrane ((d) *P* = 0.037).

Image analysis of the plasma membrane also revealed a dose-dependent increase in synaptic and extrasynaptic GABA_A_R 60 minutes after TNF*α* injection (Figures 5(g) and 5(h)) (*P* < 0.001). There was significantly more extrasynaptic GABA_A_Rs associated with the lowest TNF*α* dose relative to the middle dose (*P* < 0.003). After statistically correcting for TNF*α*-mediated changes in total GABA_A_R level with ANCOVA, there remained a significant dose-dependent effect of TNF*α* on synaptic GABA_A_R indicating that TNF*α* increases receptor trafficking to synapses on the motor neurons' somatic surface in addition to increasing extrasynaptic receptors (*P* < 0.05). The effect of TNF*α* was widespread and extended onto the contralateral side of the spinal cord (the site of vehicle injection) eliciting a dose-dependent increase in synaptic and total GABA_A_Rs at the level of the plasma membrane and the neuropil, which spanned both sides of the cord (all measures, *P* < 0.001). The extensive effect of TNF*α* on spinal tissue is also evidenced by the fact that there was no main effect of side of the cord in synaptic, nor total GABA_A_R measures (Figure 6) (all measures, *P* > 0.05). There was an interaction of dose by side for extrasynaptic GABA_A_R on the plasma membrane (*P* = 0.037), but there was not a significant interaction for any other GABA_A_R measures (*P* > 0.05) (Figure 6). Accounting for changes in total extrasynaptic GABA_A_R in the plasma membrane with ANCOVA showed no higher order interactions between dose and side for synaptic GABA_A_R (*P* > 0.05). Taken together, results indicate that there is a significant effect of TNF*α* dose that is driving GABA_A_R trafficking to synapses on the plasma membrane and that this effect was widespread throughout the tissue.

## 4. Discussion

Our present findings suggest that there is an increase in synaptic GABA_A_R that occurs in spinal cord neurons in response to TNF*α*. TNF*α* nanoinjection *in vivo *allowed us to model rapid changes in synaptic efficacy that result from the inflammatory response following SCI. Biochemical fractionation methods that have previously been shown to be sufficient to measure rapid trafficking of the excitatory AMPA receptors [13, 16] did not appear to have sufficient resolution to detect changes in inhibitory GABA_A_Rs. However, quantitative high-resolution confocal microscopy on morphologically preserved tissue sections revealed significant changes in GABA_A_Rs 60 minutes after TNF*α* injection. Analysis of the total neuropil in confocal z-series revealed significant increases in GABA_A_R positive puncta. By statistically correcting confocal images for total GABA_A_R puncta, we were able to isolate the effect of TNF*α* on synaptic GABA_A_R trafficking, revealing a specific increase in synaptic GABA_A_R levels in the neuropil. By applying algorithmic postprocessing of confocal images, we were able to generate image-based subcellular fractionation, isolating the plasma membrane of large ventral motor neurons. Analysis of this confocal subcellular fraction revealed a U-shaped TNF*α* dose-response effect on GABA_A_R levels at both extrasynaptic and synaptic sites on the motor neuron plasma membrane.

The present results contribute to a more complete understanding of TNF*α*-mediated, dynamic receptor changes at synapses following the inflammatory response in SCI. TNF*α* tissue levels have been shown to peak 60 minutes after SCI [18]. This increase in TNF*α* has potential to contribute to excitotoxic cell death in the spinal cord [11]. *In vivo *studies have shown that TNF*α* causes GluA2-lacking AMPARs to be trafficked to synapses of spinal neurons [13], thereby contributing to excitotoxicity by increasing neural permeability to Ca^++^. Inhibitory synaptic strength is directly correlated to the number of synaptic GABA_A_Rs, thus any mechanism that controls the quantity of synaptic GABA_A_Rs can have a profound effect on neuronal excitability [1–3]. Given this, our study suggests that GABA_A_R trafficking to the synapse may serve as a homeostatic mechanism that combats the excitotoxic effect of TNF*α* following SCI *in vivo*. An increase in extrasynaptic GABA_A_R as well as total GABA_A_R is consistent with research showing that GABA_A_R are first exocytosed onto the plasma membrane before trafficking laterally to synapses [7, 19]. An upregulation of extrasynaptic GABA_A_R may be necessary in order to ensure a sufficient availability of GABA_A_Rs for trafficking to synaptic sites. Increased GABA_A_R synthesis may occur in this system to meet the demands placed on intracellular receptor stores imposed by trafficking.

An unexpected finding was that extrasynaptic and total GABA_A_Rs decreased following the middle dose of TNF*α*. This phenomenon could be due to a compensatory homeostatic degradation of GABA_A_R at the middle TNF*α* dose. At a low dose, GABA_A_R synthesis could increase to maintain cellular reserves of the receptor for trafficking to the membrane, yet an increase in GABA_A_R at a low dose could be slight enough to go undetected by regulatory mechanisms that would otherwise decrease GABA_A_R accumulation [15, 17]. However, at the medium TNF*α* dose, GABA_A_R synthesis could increase to a level that would elicit a rapid homeostatic degradation or ubiquitination of these receptors so that there is a drop in total GABA_A_R protein following the middle dose. A large and rapid GABA_A_R accumulation following a high dose of TNF*α* could conceivably overwhelm homeostatic compensatory degradation of GABA_A_R resulting in a global increase in the protein.

Results also showed that at high doses of TNF*α*, there was a widespread effect of synaptic and total GABA_A_R protein that extended onto the contralateral side of the spinal cord into the region of albumin nanoinjection, as previously reported for AMPAR receptor trafficking effects [13]. At a high dose, there was not a significant difference between GABA_A_R measures on the TNF*α*-injected side and the contralateral albumin side. Yet, at lower doses, TNF*α* does not influence GABA_A_R measures at the site of albumin injection. Our results demonstrate that the TNF*α*-affected area increases with the size of the dose. This result could be explained by evidence that TNF*α* promotes a heightened inflammatory response, which causes an increase in glial activation leading to the release of a variety of inflammatory cytokines including TNF*α* [20–22]. Therefore, higher doses of TNF*α* could elicit a greater immune response leading to further release of TNF*α* through a positive feedback loop, which has been demonstrated *in vitro* [23, 24]. Additionally, at higher doses, TNF*α* elicits a higher excitatory effect in the neural circuitry at the injection site that could be transmitted across the midline of the spinal cord by commissural fibers [25, 26]. Higher doses of TNF*α* also are known to elicit a greater localized excitation which is propagated further relative to lower doses of TNF*α* [13]. Our results suggest that GABA_A_R trafficking may counteract this TNF*α*-induced excitotoxicity, so increasing the radius of excitation would likewise increase the tissue area affected by GABA_A_R trafficking.

**Figure 7:**
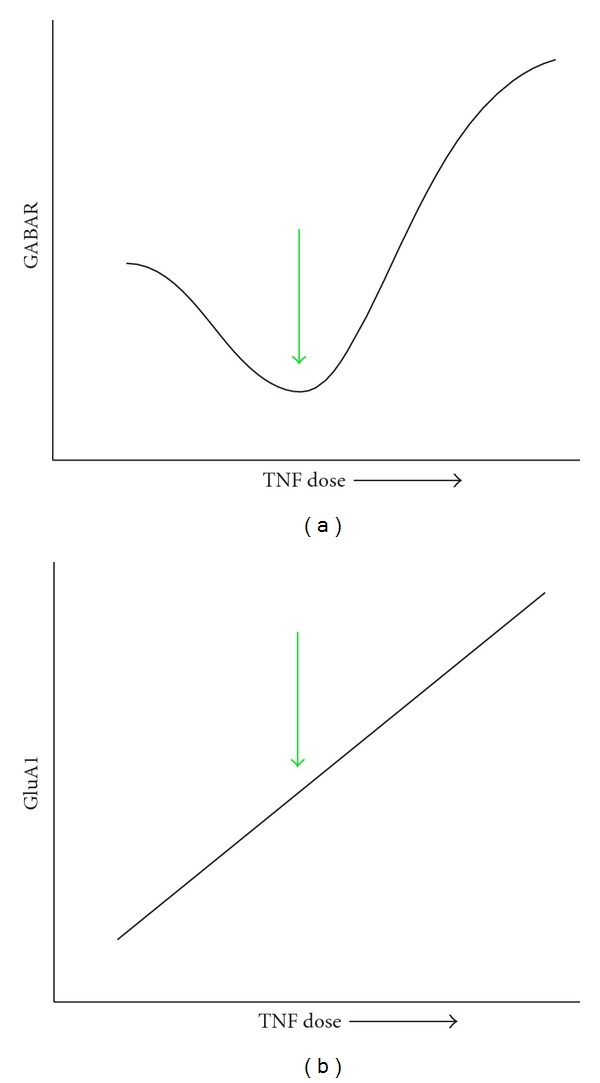
Graphical representation of combined findings from current research, Ferguson et al., 2008 [13], and Stellwagen et al., 2005 [10]. (a) Depiction of current findings that TNF causes a U-shaped response in total GABA_A_R *in vivo*. (b) Representation of findings from Ferguson et al. [13]that TNF causes an increase in total GluA1 *in vivo*. Green arrows represent the findings of Stellwagen et al., 2005 [10] that a particular dose of TNF elicits an increase in GluA1 and a decrease in GABA_A_R *in vitro*.

At first glance, our results seem to contradict the *in vitro* findings of Stellwagen et al. that TNF*α* decreases surface GABA_A_Rs through endocytosis while simultaneously increasing surface GluA2-lacking AMPARs [10]. Taken together with our prior publications, we have now found *in vivo* that GABA_A_Rs and AMPARs are both trafficked to the membrane after TNF nanoinjection (Figures 4–6) [13]. Although we found an increase in synaptic GABA_A_R following the highest TNF*α* dose, our experiment also showed that TNF*α* elicited a nonlinear dose-response effect on GABA_A_R levels (total GABA_A_R in neuropil and extrasynaptic GABA_A_R). An interesting finding was that the middle dose of TNF*α* used in our study elicited a lower amount of total GABA_A_R relative to our highest and lowest drug doses (Figure 7). This complex, nonlinear dose-dependent relationship of TNF*α* on GABA_A_R trafficking, actually replicates the finding of Stellwagen et al. when examined in the context of the study by Ferguson et al. [13], which demonstrated that, at 0.1 *μ*M TNF*α*, the very same intermediate dose used in our experiment, there was an increase in extrasynaptic GluA1 coinciding with our decrease in GABA_A_R (Figure 7). Our experiment expands upon these *in vitro *findings, recontextualizing them *in vivo* and revealing a potentially nonlinear dependence of GABA_A_R trafficking on TNF*α*. An *in vivo* account of TNF*α*-mediated GABA_A_R trafficking simulates the microenvironmental tissue changes following injury. While cell culture enables greater control over experimental variables and outcomes, a limitation of *in vitro *studies is their inability to replicate the compensatory homeostatic mechanisms present in a whole organism. In analyzing the effect of a range of TNF*α* doses on GABA_A_R trafficking, we can glean time-emergent effects following injury since TNF*α* is constitutively expressed following SCI [18]. Thus, there appear to be both dose and time components to TNF*α* receptor trafficking *in vivo*.

Further studies are necessary in order to corroborate our current findings and determine their functional consequences. It has been shown that mIPSC strength is directly correlated with the number of postsynaptic GABA_A_Rs indicating that trafficking receptors to the synapse greatly affects neuronal excitability [15]. Electrophysiological studies are critical in order to determine whether TNF*α*-mediated GABA_A_R trafficking translates into differences in synaptic current transmission. Aside from GABA_A_R trafficking, other modulatory mechanisms regulate receptor functionality and distribution including changes in GABA_A_R phosphorylation, half-life, ubiquitination, lysosomal degradation, and interactions with other glutamate receptor subtypes such as the NMDA receptor [6, 7, 17, 27, 28]. An additional physiological variable to consider is that, under certain conditions, GABAergic synapses can be excitatory. GABA_A_Rs are permeable to both Cl^−^ and HCO3^−^ and these currents have reversal potentials (E_GABA_) close to the neuronal resting potential. Physiological conditions that cause the membrane potential to become more negative than E_GABA_ reverse the direction of ion flow through GABA_A_Rs [29]. For example, a heightened level of neuronal activity has been shown to transiently alter E_GABA_ in hippocampal CA1 pyramidal cells *in vitro.* GABAergic synapses become depolarizing after a high-frequency train of stimulation causing an accumulation of Cl^−^ inside the cell and high K^+^ outside, in the interstitial fluid [30, 31]. In the spinal cord, excitatory effects of GABA_A_Rs have been implicated in pathological pain conditions and maladaptive spinal plasticity [32–34]. In light of these caveats, it is essential to determine the electrophysiological consequences of TNF*α*-mediated GABA_A_R trafficking. A better understanding of the link between TNF*α*-induced synaptic GABA_A_R trafficking and neural excitability is a clear target for further research that may lead to a novel therapeutic target for combating the spread of excitotoxicity after SCI and other CNS diseases.
